# Cardiovascular Health Among Non‐Hispanic Asian Americans: NHANES, 2011–2016

**DOI:** 10.1161/JAHA.118.011324

**Published:** 2019-06-26

**Authors:** Jing Fang, Zefeng Zhang, Carma Ayala, Angela M. Thompson‐Paul, Fleetwood Loustalot

**Affiliations:** ^1^ Division for Heart Disease and Stroke Prevention National Center for Chronic Disease Prevention and Health Promotion Centers for Disease Control and Prevention Atlanta GA

**Keywords:** cardiovascular health, non‐Hispanic Asian American, race and ethnicity, surveillance, Epidemiology, Lifestyle, Race and Ethnicity

## Abstract

**Background:**

Asian Americans are the fastest growing population in the United States, but little is known about their cardiovascular health (CVH). The objective of this study was to assess CVH among non‐Hispanic Asian Americans (NHAAs) and to compare these estimates to those of non‐Hispanic white (NHW) participants.

**Methods and Results:**

Merging NHANES (National Health and Nutrition Examination Survey) data from 2011 to 2016, we examined 7 metrics (smoking, weight, physical activity, diet, blood cholesterol, blood glucose, and blood pressure) to assess CVH among 5278 NHW and 1486 NHAA participants aged ≥20 years. We assessed (1) the percentage meeting 6 to 7 metrics (ideal CVH), (2) the percentage meeting only 0 to 2 metrics (poor CVH), and (3) the overall mean CVH score. We compared these estimates between NHAAs and NHWs and among foreign‐born NHAAs by birthplace and number of years living in the United States. The adjusted prevalence of ideal CVH was 8.7% among NHAAs and 5.9% among NHWs (*P*<0.001). NHAAs were significantly more likely to have ideal CVH (adjusted prevalence ratio: 1.42; 95% CI, 1.29–1.55) compared with NHWs. Among NHAAs, there was no significant difference in ideal CVH between US‐ and foreign‐born participants, nor by number of years living in the United States. With lower body mass index thresholds (<23, normal weight) for NHAAs, there were no statistically significant differences in the adjusted prevalence of ideal CVH (6.5% versus 5.9%, *P*=0.216) between NHAAs and NHWs.

**Conclusions:**

NHAAs had a higher prevalence of overall ideal CVH compared with NHWs. However, when using a lower body mass index threshold for NHAAs, there was no difference in ideal CVH between the groups.

**Clinical Perspective**

**What Is New?**

- When using standard thresholds for body mass index, non‐Hispanic Asian Americans (NHAAs) had a higher percentage with ideal cardiovascular health (CVH) and a lower percentage with poor CVH compared with non‐Hispanic white Americans.
- When a lower body mass index threshold was applied to NHAAs, no difference in ideal and poor CVH was detected.
- Among NHAAs, country of birth and number of years living in the United States were not related to CVH.

**What Are the Clinical Implications?**

- The low levels of ideal CVH among both NHAA and non‐Hispanic white participants suggest that public health and clinical practice have ample opportunities to support improvement of health behaviors and outcomes that support overall CVH.
- Caution should be taken when applying standard thresholds to diverse populations. In this study, significant differences in ideal CVH between NHAA and non‐Hispanic white participants were negated when Asian‐specific body mass index cut points, which are lower than the cut points for normal weight among the general population, were applied.
- These findings highlight the need for the application of population‐specific and culturally appropriate metrics when assessing CVH.

## Introduction

Asian Americans are one of the fastest growing populations in the United States. The 2010 Census data showed that ≈5% of total US population was Asian American.^1^ The Asian American population is projected to double from 18 million (2016) to 37 million by 2060.^2^ The projected increase of the Asian population in the United States will require additional focus on the health conditions within this population.^3^ In general, Asian Americans reportedly face proportionately greater mortality related to hypertensive heart disease and cerebrovascular disease, especially intracerebral hemorrhage, compared with non‐Hispanic white (NHW) Americans.^4^ An understanding the unique burden of cardiovascular disease and related risk factors among Asian Americans is needed.

Among people immigrating to the United States, those classified as *foreign‐born* in epidemiologic studies have commonly reported better health than those born in the United States. This association generally lessens with increased number of years living in the United States. For example, a recent report on Asian Americans with hypertension found that those born in the United States were more likely to have hypertension than those identified as foreign‐born. In addition, among those classified as foreign‐born, people who reported living in the United States for >10 years were more likely to have hypertension than recent immigrants (<5 years).^5^ This relationship has also been observed to some degree among primary cardiovascular disease outcomes. Another recent study found that rates of stroke and coronary heart disease were lower among foreign‐born Asian participants compared with those born in the United States, but no difference in prevalence was noted when assessing number of years living in the United States.^6^ Exposure, culture, and access are all important factors influencing health and health outcomes. Birthplace and duration of residency in the United States serve as limited proxies for these factors and are included in these analyses to acknowledge their influence and provide context for interpreting findings.

In 2010 the American Heart Association published criteria describing cardiovascular health (CVH).^7^ CVH has been defined in different ways depending on the data available, but it generally includes an assessment based on current recommendations for primary risk factors and behaviors (not smoking; normal weight; adequate physical activity; healthy diet; healthy blood cholesterol, blood glucose, and blood pressure).^7^ In response, numerous studies have assessed CVH among diverse populations.^8^, ^9^, ^10^, ^11^, ^12^, ^13^, ^14^ Self‐reported national data have been used to assess CVH in a combined group of Asian Americans, Pacific Islanders, and Native Hawaiians.^11^ Although Asians, Pacific Islanders, and Native Hawaiians were found to have better CVH than other racial and ethnic groups,^11^ other research has shown that health status and cardiovascular risk factors differ between Asians and Pacific Islanders.^15^ Furthermore, hypertension has been reported to be lower among Asians than among Pacific Islanders and Native Hawaiians.^16^ To our knowledge, no studies have assessed CVH metrics among Asian Americans (separately from Pacific Islanders and Native Hawaiians) using directly measured data. Since 2011, NHANES (National Health and Nutrition Examination Survey) oversampled Asian Americans, which allowed for the calculation of national estimates of health indicators for this population.^17^ The objectives of this report were to compare (1) the CVH of non‐Hispanic Asian Americans (NHAAs) and NHWs and (2) CVH among NHAAs by birthplace and number of years living in the United States.

## Methods

NHANES is a nationally representative cross‐sectional survey, conducted in 2‐year cycles, of the nonmilitary noninstitutionalized US population and uses a complex multistage‐probability sampling design. All data and materials have been made publicly available at the National Center for Health Statistics website.^18^ The survey collects self‐reported and directly measured information from participants on health conditions, behaviors, dietary intake, physical examination, and laboratory tests. NHANES has been described in detail previously.^18^ Historically, NHANES oversampled certain populations (eg, Mexican Americans), which allowed for increased reliability and precision of health status indicator estimates for these groups.^19^ Beginning in 2011, NHANES integrated methods to oversample Asians.^17^ The process included recruiting staff who were fluent in English and an Asian language. Cultural competency training was conducted with the staff. Survey materials (eg, brochures, reminder letters, consent forms, safety guidelines, instructions) were translated into selected Asian languages (eg, Chinese, Korean, Vietnamese, etc); however, because of its complexity, the questionnaire was not translated. Interpreters were available as needed.

This report includes 3 NHANES survey cycles (2011–2012, 2013–2014, and 2015–2016) among participants aged ≥20 years. *Asian* was defined using the question, “What race do you consider yourself to be?” The *Asian* category included all people having origins in the Far East, Southeast Asia, or the Indian subcontinent, including Cambodia, China, India, Japan, Korea, Malaysia, Pakistan, the Philippine Islands, Thailand, and Vietnam. A separate response was provided for *Native Hawaiian/Pacific Islander*, which allowed for the exclusion of this category in these analyses.^20^ Hispanic ethnicity was assessed using the question, “Do you consider yourself to be Hispanic or Latino?” Because 2010 US Census data identified 98.6% of the total Asian population as NHAAs,^21^ NHAAs alone were used in this report for comparison to NHWs. Participants who selected multiple races were excluded (<4%). The final 2 groups were NHAAs and NHWs. Among NHAAs, country of origin was assessed using the question, “In what country were you born?” Among those born outside of the United States (ie, foreign‐born), number of years living in United States was ascertained and categorized as <10, 10 to 19, or ≥20 years.

CVH was based on 7 metrics defined using current recommendations for smoking, weight, physical activity, diet, blood cholesterol, blood glucose, and blood pressure. *Ideal* smoking status was defined as having never smoked or having not smoked for at least 1 year. Body mass index (BMI) was obtained using directly measured height and weight (kg/m^2^), with ideal weight being defined as a BMI between 18.5 and 24.9. However, previous studies have found that the standard BMI cut point for normal weight may not be appropriate for Asian populations.^22^ To address this concern, we conducted additional analyses using an Asian‐specific cut point for ideal weight (BMI: 18.5–22.9).^23^ Physical activity was assessed through questions recording the frequency and duration of activities over the past week or month, including moderate‐ and vigorous‐intensity activities. Ideal physical activity was defined as ≥150 minutes of moderate‐intensity activities per week, ≥75 minute of vigorous‐intensity activities per week, or an equivalent combination of both.^24^ Ideal diet intake was based on the Healthy Eating Index (HEI2010), which is a measure of diet quality based on 2010 Dietary Guidelines.^25^ HEI2010 included 12 components, of which 9 support diet adequacy (total fruit, whole fruit, total vegetables, greens and beans, whole grains, dairy, total protein foods, seafood and plant proteins, and fatty acids) and 3 should be consumed in moderation (refined grains, sodium, and empty calories). The HEI2010 total score was derived from the summation of the individual component scores. Using previously published thresholds, a total score <51 was categorized as a *poor* diet, 51 to 80 indicated a *moderately healthy* diet, and ≥81 was an *ideal* diet.^25^ Fasting blood samples were used to assess blood glucose and cholesterol. Ideal blood glucose was defined as fasting blood glucose ≤100 mg/dL or HbA1c <5.7% without medication, and ideal blood cholesterol was defined as total cholesterol <200 mg/dL without medication.^26^ Blood pressure measurements were taken using standardized protocols. Three consecutive blood pressure readings were obtained and the average measurement of the three was used. Ideal blood pressure was defined as <120/80 mm Hg without using antihypertensive medication. Participants with 6 or 7 ideal CVH metrics, using the previously described standards, were classified as having ideal CVH. Participants with ≤2 ideal CVH metrics were classified as having poor CVH. The outcome measurements were (1) percentage of participants with ideal CVH; (2) percentage of participants with poor CVH; and (3) mean CVH score, which was defined as the mean score of the total summated CVH metrics giving each metric equal weight.

Basic demographic characteristics including age, sex, level of education, and health insurance status were selected as covariates because prior evidence has demonstrated CVH disparities among these factors.^11^, ^14^ Age was categorized as *20 to 44, 45 to 64*, and *≥65* years; sex as *male* and *female*; level of education as *less than high school, high school graduate*, and *more than high school*; and health insurance as *any health insurance* or *none*.

The 3 combined NHANES cycles had a total of 2151 NHAAs and 6376 NHWs who were aged ≥20 years. Participants were excluded from the primary sample if they were pregnant (n=83), were missing information on demographic characteristics (n=6), and were missing any information on the CVH metrics, including smoking status (n=83), BMI (n=71), physical activity (n=3), dietary intake (n=1078), blood cholesterol (n=294), blood glucose (n=216), and blood pressure (n=181). The total sample excluded for missing data was 1763 (some excluded participants were missing information in multiple areas). The final analytic sample was 6764, including 1486 NHAAs and 5278 NHWs.

NHANES protocols were approved annually by the Centers for Disease Control and Prevention's (CDC's) National Center for Health Statistics Research Ethics Review Board. Informed consent was obtained from all participants.

### Statistical Analyses

Data on characteristics were expressed as means and standard errors for continuous variables or as percentages for categorical variables. We used χ^2^ tests to compare differences in baseline percentage and adjusted percentage (adjusted for age, sex, level of education, and health insurance status) of ideal and poor CVH between NHAAs and NHWs and among NHAAs by birthplace and number of years living in the United States. We used Student *t* tests to compare differences in baseline mean age and the adjusted mean CVH score for NHAAs and NHWs and by birthplace and number of years living in the United States among foreign‐born NHAAs. We used logistic regression models to estimate the prevalence ratio and 95% CIs of ideal and poor CVH of NHAAs, using NHWs as the referent, adjusting for age, sex, level of education, and insurance status.^27^ We used multinomial logistic regression to assess the prevalence ratio and 95% CIs of ideal and poor CVH among NHAAs using US‐born as the referent. Additional analyses were conducted to compare the adjusted percentages of normal weight, ideal or poor CVH, and prevalence ratios between NHAAs and NHWs and by birthplace and years of living in the United States among US‐ and foreign‐born NHAAs, using a previously defined Asian‐specific BMI cut point (BMI: 18.5–22.9) to define normal weight. Statistical analyses were performed using SUDAAN v11 (RTI International) to take the complex sampling design into account. All tests of statistical significance were 2‐tailed, and a probability value <0.05 was considered significant.

## Results

Compared with NHWs, NHAAs were significantly younger (44.5% versus 49.9%, *P*<0.001), had higher levels of educational attainment (76.4% versus 70.0% with more than high school education, *P*<0.001), and were less likely to have health insurance (84.1% versus 88.5%, *P*=0.023). Among NHAAs, foreign‐born adults who reported living in the United States for ≥10 years were older than US‐born adults or those living in the United States for <10 years. US‐born NHAAs had higher education attainment than foreign‐born NHAAs. The percentage of individuals reporting health insurance was lowest among foreign‐born NHAAs who had lived in the United States for <10 years (74.4%) and highest among those living in the United States for ≥20 years (90.7%; Table 1).

**Table 1:** Demographic Characteristics of NHAAs and NHWs and Among NHAAs by Birthplace and Number of Years of Living in the United States, NHANES, 2011–2016

Demographic Characteristics	NHW (n=5278)	NHAA (n=1486)	*P* Value^a^	NHAA Subgroup				
				US‐Born (n=214)	Foreign‐Born, in USA <10 y (n=367)	Foreign‐Born, in USA 10–19 y (n=309)	Foreign‐Born, in USA ≥20 y (n=586)	*P* Value^a^
Age, y								
Mean (SE)	49.9 (0.45)	44.5 (0.82)	<0.001	35.2 (1.64)	35.2 (1.20)	42.8 (1.44)	54.0 (0.81)	<0.001
% (SE)								
20–44	38.3 (1.29)	52.8 (2.07)	<0.001	75.6 (4.35)	81.1 (2.90)	61.8 (4.97)	24.3 (2.10)	<0.001
45–64	39.4 (1.00)	32.9 (1.51)		16.3 (3.37)	13.4 (2.34)	29.5 (3.38)	51.5 (2.62)	
≥65	22.3 (0.88)	14.4 (1.44)		8.1 (1.87)	5.5 (1.52)	8.8 (2.45)	24.2 (2.23)	
Sex, % (SE)								
Men	49.4 (0.67)	47.6 (1.24)	0.235	50.8 (4.23)	53.7 (2.32)	46.0 (3.39)	43.7 (1.96)	0.010
Women	50.6 (0.67)	52.4 (1.24)		49.2 (4.23)	46.3 (2.32)	54.0 (3.39)	56.3 (1.96)	
Education, % (SE)								
Less than high school	9.1 (0.98)	10.4 (1.47)	<0.001	3.2 (1.27)	10.7 (1.95)	10.6 (1.87)	12.6 (2.28)	<0.001
High school	20.9 (1.14)	13.3 (1.35)		12.0 (3.37)	13.9 (3.20)	16.7 (3.30)	11.9 (1.69)	
More than high school	70.0 (1.81)	76.4 (2.02)		84.9 (3.47)	75.4 (3.64)	72.7 (3.44)	75.6 (2.82)	
Health insurance, % (SE)								
Yes	88.5 (0.81)	84.1 (1.84)	0.023	82.7 (4.48)	74.4 (3.90)	83.0 (2.43)	90.7 (1.64)	<0.001
No	11.5 (0.81)	15.9 (1.84)		17.3 (4.48)	25.6 (3.90)	17.0 (2.43)	9.3 (1.64)	

NHAA indicates non‐Hispanic Asian American; NHANES, National Health and Nutritional Examination Survey; NHW, non‐Hispanic white.

a *P* values were assessed with Student *t* tests using PROC DESCRIPT for age and with χ^2^ tests using PROC CROSSTAB for the percentage among NHAA groups or between NHW and NHAA groups.

Adjusted percentages for the 7 individual CVH metrics for NHAAs and NHWs and among NHAAs by birthplace and number of years of living in the United States are presented in Table 2. Compared with NHWs, NHAAs were more likely to be nonsmokers (90.4% versus 78.5%, *P*<0.001), to have normal weight (55.9% versus 29.7%, *P*<0.001), to report a healthy diet (5.03% versus 2.72%, *P*<0.001), and to have normal blood pressure (44.0% versus 40.1%, *P*=0.004). However, NHAAs were less likely to have normal blood glucose (61.4% versus 73.5%, *P*<0.001). No differences were observed between NHAAs and NHWs meeting physical activity recommendations and having normal blood cholesterol. Among NHAAs, US‐born NHAAs had the highest percentage meeting physical activity recommendations (44.4%), and foreign‐born NHAAs living in the United States <10 years had the lowest percentage (33.6%). In addition, US‐born NHAAs had the highest percentage of normal blood glucose (68.0%), and foreign‐born NHAAs living in the United States for 10 to 19 years had the lowest (57.0%). However, the percentage of normal blood pressure was highest among foreign‐born NHAAs living in the United States for <10 years (52.4%) and lowest among US‐born NHAAs (36.4%).

**Table 2:** Individual CVH Metrics and Overall CVH Among NHAAs and NHWs, NHANES 2011–2016

Measures	NHW	NHAA	*P* Value^a^	NHAA Subgroup				
				US‐Born	Foreign‐Born, in USA <10 y	Foreign‐Born, in USA 10–19 y	Foreign‐Born, in USA ≥20 y	*P* Value^a^
Total, n	5278	1486		214	367	309	586	
Individual CVH metrics								
Does not smoke	78.5 (0.98)	90.4 (0.85)	<0.001	86.1 (1.68)	92.7 (1.31)	90.7 (1.69)	89.9 (1.41)	0.515
Normal weight (BMI <25)	29.7 (0.84)	55.9 (1.47)	<0.001	48.9 (4.09)	57.4 (2.70)	58.4 (2.98)	56.2 (2.47)	0.151
Recommended physical activity	40.9 (1.18)	39.0 (1.64)	0.263	44.4 (2.92)	33.6 (2.64)	35.9 (2.99)	42.4 (2.52)	0.045
Healthy diet	2.72 (0.30)	5.03 (0.45)	<0.001	4.54 (1.43)	5.01 (1.65)	6.67 (1.59)	4.54 (0.72)	0.594
Normal blood cholesterol	54.1 (1.10)	55.1 (1.83)	0.567	58.1 (4.02)	56.6 (3.76)	53.9 (2.80)	53.3 (2.38)	0.415
Normal glucose	73.5 (0.76)	61.4 (1.59)	<0.001	68.0 (4.67)	67.9 (2.78)	57.0 (2.84)	59.1 (1.98)	0.007
Normal blood pressure	40.1 (1.00)	44.0 (1.21)	0.004	36.4 (2.86)	52.4 (2.90)	45.9 (2.15)	40.5 (2.19)	<0.001
CVH								
Ideal	5.9 (0.54)	8.7 (0.77)	<0.001	8.2 (1.23)	10.4 (1.48)	6.9 (1.28)	8.3 (1.37)	0.615
Poor	33.5 (0.99)	26.3 (1.42)		28.5 (3.28)	22.5 (2.50)	27.0 (2.54)	27.1 (2.00)	
CVH score, mean (SE)	3.19 (0.03)	3.49 (0.04)	<0.001	3.51 (0.10)	3.63 (0.08)	3.46 (0.09)	3.42 (0.06)	0.236

Multinomial logistic regression was used to estimate the adjusted percentage of individual ideal CVH metrics and overall CVH among 4 NHAAs groups. Logistic regression was used to estimate the adjusted percentage for NHWs and NHAAs, adjusted for age, sex, level of education, and insurance status. Participants with 6 or 7 ideal CVH metrics were classified as having ideal CVH, and those with ≤2 ideal CVH metrics were classified as having poor CVH. Data are shown as % (SE) except as noted. BMI indicates body mass index; CVH, cardiovascular health; NHAA, non‐Hispanic Asian American; NHANES, National Health and Nutritional Examination Survey; NHW, non‐Hispanic white.

a *P* values were assessed with the Student *t* test for mean CVH scores and with the χ^2^ test for adjusted percentage.

In addition, Table 2 presents the adjusted percentages of ideal CVH (6–7 metrics) and poor CVH (0–2 metrics) and the mean CVH score for NHAAs and NHWs and among NHAAs by birthplace and number of years of living in the United States. NHAAs had a significantly higher percentage of ideal CVH (8.7% versus 5.9%), a lower percentage of poor CVH (26.3% versus 33.5%), and a significantly higher mean score (3.49 versus 3.19) than NHWs (all *P*<0.001). Among NHAAs, no differences were noted by birthplace or number of years of living in the United States for ideal CVH, poor CVH, or mean CVH score.

Using logistic regression models, we assessed the prevalence ratio of ideal CVH and poor CVH between NHAAs and NHWs after adjusting for age, sex, level of education, and insurance status (Table 3). Using NHWs as the referent, NHAAs were 42% more likely to have ideal CVH and 24% less likely to have poor CVH. Birthplace and number of years living in the United States were not associated with ideal or poor CVH when the analyses were limited to NHAAs only.

**Table 3:** Adjusted Prevalence Ratio (95% CI) of Ideal and Poor CVH Among NHAAs and NHWs, NHANES 2011–2016

Measures	NHW	NHAA	NHAA Subgroup			
			US‐Born	Foreign‐Born, in USA <10 y	Foreign‐Born, in USA 10–19 y	Foreign‐Born, in USA ≥20 y
Total, n	5278	1486	214	367	309	586
Ideal CVH	1.00	1.42 (1.29–1.55)	1.00	1.10 (0.96–1.25)	1.01 (0.90–1.14)	0.96 (0.84–1.10)
Poor CVH	1.00	0.76 (0.69–0.85)	1.00	0.83 (0.62–1.12)	1.05 (0.77–1.43)	1.13 (0.85–1.51)

Multinomial logistic regression was used to estimate the adjusted prevalence ratio of ideal or poor CVH comparing each foreign‐born Asian group with US‐born NHAAs. Logistic regression was used to estimate the adjusted prevalence ratio comparing NHAAs to NHWs, adjusting for age, sex, level of education, race/ethnicity and insurance status. Participants with 6 or 7 ideal CVH metrics were classified as having ideal CVH, and those with ≤2 ideal CVH metrics were classified as having poor CVH. CVH indicates cardiovascular health; NHAA, non‐Hispanic Asian American; NHANES, National Health and Nutritional Examination Survey; NHW, non‐Hispanic white.

Using a lower cut point for normal BMI among NHAAs changed the relationships among the groups. Using a normal‐weight cut point of 18.5 to 22.9 for NHAAs and a standard cut point for NHWs, NHAAs continued to have a higher percentage of normal weight (34.2% versus 29.7%, *P*=0.007). However, there were no significant differences in the prevalence of ideal CVH (6.5% versus 5.9%) and poor CVH (31.2% versus 33.5%) or in mean CVH score (3.27 versus 3.19) between NHAAs and NHWs (Table 4). Among NHAAs by birthplace and number of years living in the United States, no significant differences were noted in the prevalence of ideal and poor CVH or in mean CVH. There was no difference in the adjusted prevalence ratios of ideal CVH between NHAAs and NHWs or among NHAAs by birthplace and number of years of living in the United States (Table 4).

**Table 4:** Adjusted Prevalence of Normal Weight, Ideal and Poor CVH, and Mean CVH Score and Adjusted Prevalence Ratio of Ideal and Poor CVH Using Asian‐Specific Normal‐Weight Criteria (BMI <23) for NHAAs and Standard Cut Point (BMI <25) for NHWs, NHANES 2011–2016

Sample Characteristics	NHW	NHAA	*P* Value^a^	NHAA Subgroup				
				US‐Born	Foreign‐Born, in USA <10 y	Foreign‐Born, in USA 10–19 y	Foreign‐Born, in USA ≥20 y	*P* Value^a^
Adjusted prevalence, % (SE)								
Normal weight	29.7 (0.84)	34.2 (1.56)	0.007	31.3 (3.86)	35.9 (2.83)	32.9 (2.41)	34.9 (1.90)	0.599
Ideal CVH	5.9 (0.54)	6.5 (0.66)	0.216	5.9 (1.12)	7.4 (1.12)	5.6 (1.09)	6.4 (1.37)	0.285
Poor CVH	33.5 (0.99)	31.2 (1.58)		32.5 (3.99)	26.7 (2.80)	32.1 (2.14)	32.6 (2.10)	
Mean CVH Score	3.19 (0.03)	3.27 (0.04)	0.093	3.32 (0.10)	3.43 (0.08)	3.17 (0.09)	3.21 (0.06)	0.204
Adjusted prevalence ratio (95% CI)								
Ideal CVH	1.00	1.12 (0.87–1.44)		1.00	1.06 (0.68–1.65)	0.71 (0.38–1.31)	0.92 (0.50–1.69)	
Poor CVH	1.00	0.92 (0.83–1.02)		1.00	0.85 (0.64–1.13)	1.05 (0.79–1.40)	1.10 (0.83–1.46)	

Multinomial logistic regression was used to estimate the adjusted percentage of individual ideal cardiovascular health metrics and overall cardiovascular health among 4 NHAAs groups. Logistic regression was used to estimate the adjusted percentage comparing NHWs and NHAAs, adjusted for age, sex, education, and insurance status. Multinomial logistic regression was used to estimate the adjusted prevalence ratio of ideal or poor CVH comparing each foreign‐born Asian group with US‐born Asians. Logistic regression was used to estimate the adjusted prevalence ratio comparing NHAAs and NHWs, adjusting by age, sex, education, and insurance status. Participants with 6 or 7 ideal CVH metrics were classified as having ideal CVH, and those with ≤2 ideal CVH metrics were classified as having poor CVH. BMI indicates body mass index; CVH, cardiovascular health; NHAA, non‐Hispanic Asian American; NHANES, National Health and Nutritional Examination Survey; NHW, non‐Hispanic white.

a *P* value was assessed with the Student *t* test for mean CVH scores and with the χ^2^ test for adjusted percentage.

## Discussion

In this nationally representative sample, NHAAs had a higher percentage of ideal CVH, a lower percentage of poor CVH, and better overall CVH scores (represented by higher mean CVH score) compared with NHWs. Factors related to a favorable CVH score included younger age, female sex, and a higher level of education. Among NHAAs, birthplace and number of years of living in the United States were not related to CVH scores. However, when a lower threshold for BMI was applied to NHAAs, a higher percentage of the group continued to be classified as normal weight than NHWs, but the significant differences in ideal and poor CVH between the groups was negated.

Previous studies have consistently shown that ideal CVH is uncommon in the United States. A previous report using data from the ARIC (Atherosclerosis Risk in Communities) study, for example, showed that only about 0.1% of participants had ideal CVH.^8^ A comparable report using NHANES data found that the percentages of the population with ideal CVH were 2.0%, 1.3%, and 1.2% in the study periods 1988–1994, 1999–2004, and 2005–2010, respectively.^26^ However, these studies did not report NHAAs as a single group because of small sample sizes. Although the BRFSS (Behavioral Risk Factor Surveillance System), a state‐based telephone survey conducted in all 50 states, reported ideal CVH among Asians, Pacific Islanders, and Native Hawaiians as a single group,^11^ there are likely significant differences between Asians and Pacific Islanders/Native Hawaiians, either culturally or physiologically, that may affect CVH scores.^28^ To our knowledge, this report is the first to assess the CVH status of NHAAs in a nationally representative sample.

Among individual CVH metrics, we found most metrics showed a more favorable distribution for NHAAs than NHWs. Significantly higher percentages of NHAAs, for example, did not smoke, were of normal weight, reported a healthy diet, and had normal blood pressure compared with NHWs. There were no differences in the percentage of adults who reported meeting physical activity recommendations or who had normal blood cholesterol between NHAAs and NHWs. However, NHWs had a higher percentage with normal blood glucose compared with NHAAs, a result that has also been reported in other studies.^29^ For example, Menke and colleagues reported that the age‐standardized prevalence of total diabetes mellitus was higher among NHAAs compared with NHWs (20.6% versus 11.3%, *P*=0.007).^30^ This finding suggests that diabetes mellitus prevention and clinical treatment to control diabetes mellitus may be important initial intervention targets for NHAAs.

Obesity has been associated with increased cardiovascular morbidity and mortality.^31^ Previous studies have shown that Asian populations have increased risk of diabetes mellitus and cardiovascular risk factors (eg, hypertension and high blood cholesterol) at lower BMI thresholds.^32^ Using standard BMI cut points for overweight and obesity among Asian populations may fail to identify some individuals who are at increased cardiovascular risk. The World Health Organization/International Association for the Study of Obesity/International Obesity Task Force proposed the use of a BMI cut point <23 to classify normal weight in Asian populations.^33^ In this report, we found that the percentage of normal weight was significantly higher among NHAAs compared with NHWs using both the standard normal‐weight cut point of <25 (55.9% versus 29.7%) and the Asian‐specific normal‐weight cut point of <23 (34.2% versus 29.7%). However, the statistically significant differences between NHAAs and NHWs in ideal and poor CVH observed using the standard BMI cut point (<25) were not observed using the lower BMI threshold (<23) for NHAAs. The difference in ideal CVH among NHAAs using the standard normal‐weight cut point (8.7%) and the Asian‐specific cutoff point (6.5%) was 2.2%. If these estimates were extrapolated to the total US NHAAs population, an estimated 390 000 NHAAs (total 18 million Asian population in 2016, 98.5% of NHAAs) would be potentially misclassified as having ideal CVH using the standard cut point rather than a lower threshold for BMI in this subgroup.

This study has several limitations. The measures of smoking, physical activity, and dietary intake from NHANES were based on self‐reported data and were subject to recall bias. However, prior studies have demonstrated the validity of self‐assessment among these factors (eg, smoking status,^34^ dietary intake^35^), with physical activity estimates varying depending on the mode of assessment (self‐reported versus objectively measured^36^). Because of the low percentage of the population with ideal CVH (<1% with all 7 metrics), those meeting 6 to 7 CVH metrics were classified as having ideal CVH. Last, given the relatively small sample of NHAAs, we were unable to assess CVH among Asian subgroups (eg, Chinese, Japanese, Asian Indian). Nevertheless, this report is the first to assess CVH among NHAAs by using a nationally representative sample.

This report highlights potential challenges in applying standard CVH criteria to diverse populations while acknowledging persistent low levels of CVH in the United States. Improving the CVH of all Americans while reducing deaths from heart disease and stroke is a national priority,^37^ and multiple public health and clinical partners are striving to support these goals. For example, the CDC supports cardiovascular disease prevention efforts in all 50 states and the District of Columbia and coleads with Centers for Medicare and Medicaid Services the Million Hearts initiative, which prioritizes population‐level interventions and provides resources to support evidenced‐based interventions across diverse settings and populations to prevent heart attacks and strokes.^38^ The American Heart Association, the American Medical Association, and other organizations have national initiatives targeting diverse stakeholders in the clinical and public health arenas that are advancing CVH in the United States.

In conclusion, standard cut points for well‐recognized cardiovascular disease risk factors may not be appropriate for all populations. In this study, although ideal CVH appeared to be more prevalent among NHAAs, the use of an Asian‐specific BMI threshold mitigated this difference. Low levels of ideal CVH have been noted frequently across diverse populations in the United States, and population‐specific and culturally appropriate interventions across varied settings may be needed to produce substantive improvement in CVH.

## Disclosures

None.
